# Profiling Bioactive Components of Natural Eggshell Membrane (NEM) for Cartilage Protection and Its Protective Effect on Oxidative Stress in Human Chondrocytes

**DOI:** 10.3390/ijms252011304

**Published:** 2024-10-21

**Authors:** Jin-Woo Kim, Dong-Ho Lee, Kang-Woo Lee, In-Su Na, Na-Yeon Lee, Jong-Kyu Kim, Yoon-Seok Chun, Nam-Kyu Yoon, Byung-Kwon Kim, Sung-Keun Yang, Soon-Mi Shim

**Affiliations:** 1 Department of Food Science and Biotechnology, Sejong University, Neungdong-ro 209, Gwangjin-gu, Seoul 05006, Republic of Korea; kimjinu0915@naver.com (J.-W.K.); eastlight1221@naver.com (D.-H.L.); lkangwoo12@naver.com (K.-W.L.); nis9909@naver.com (I.-S.N.); dlskdus53790@naver.com (N.-Y.L.); 2 ARI BnC Co., Ltd., 1F, 16-6 Eondong-ro 125, Giheung-gu, Yongin-si 16985, Republic of Korea; jkkim@aribnc.com (J.-K.K.); ceochun@aribnc.com (Y.-S.C.); nkyoon@aribnc.com (N.-K.Y.); kimbk8209@aribnc.com (B.-K.K.); 3 Sung Dong Bio Co., Ltd., 1311, Seonyudo Tewnty First Valley, 157 Yangpyeongro, Youngdeungpo-gu, Seoul 07207, Republic of Korea; skyang3517@gmail.com

**Keywords:** natural eggshell membrane (NEM), oxidative stress, peptides, human chondrocytes (SW-1353), osteoarthritis (OA)

## Abstract

The current study aimed to investigate the physicochemical properties of the natural eggshell membrane (NEM) and its protective effects against H_2_O_2_-induced oxidative stress in human chondrocytes (SW-1353). Bioactive components from NEM related to cartilage were profiled, consisting of 1.1 ± 0.07% hyaluronic acid, 1.2 ± 0.25% total sulfated glycosaminoglycans as chondroitin sulfate, 3.1 ± 0.33% collagen, and 54.4 ± 2.40% total protein. Protein was hydrolyzed up to 43.72 ± 0.76% using in vitro gastro–intestinal digestive enzymes. Peptides eluted at 9.58, 12.46, and 14.58 min using nano-LC-ESI-MS were identified as TEW, SWVE, and VYL peptides with an M/Z value of 435.1874, 520.2402, and 394.2336, respectively. Radical scavenging activity of NEM at 10 mg/mL using the ABTS assay was revealed to be 2.1 times higher than that of the positive control. NEM treatment significantly enhanced cellular SOD expression (*p* < 0.05). Pre-treatment with NEM (0.1, 1, and 10 mg/mL) dose-dependently reduced H_2_O_2_-induced ROS levels in SW-1353. Cell live imaging confirmed that NEM pre-treatment led to a significant reduction in apoptosis expression compared to control. Results from the present study suggest that NEM rich in cartilage protective components including hyaluronic acid, collagen, and chondroitin antioxidative peptides could be a potential therapeutic agent for osteoarthritis (OA) by scavenging oxidative stress.

## 1. Introduction

Osteoarthritis (OA) is a chronic degenerative joint disorder characterized by the progressive breakdown of articular cartilage, primarily driven by oxidative stress in chondrocytes [1]. Oxidative stress resulting from the excessive production of reactive oxygen species (ROS) is known to induce cellular damage, protein degradation, and DNA mutations that can contribute to OA progression [2,3]. Particularly, the accumulation of ROS in chondrocytes disrupts cellular homeostasis and accelerates cartilage degeneration by inducing upregulation of matrix-degrading enzymes and pro-inflammatory cytokines [4,5]. Consequently, the removal of ROS and the prevention of oxidative damage are suggested to be critical therapeutic strategies for OA [6,7,8].

Natural compounds, including ascorbic acid, vitamin E, glutathione, and plant-derived polyphenols, have been identified as effective ROS scavengers with the potential to mitigate OA-related oxidative stress [9,10]. Polyphenols, known for their potent antioxidant properties, modulated several key signaling pathways that played crucial roles in cellular processes. For example, the signal transducer and activator of the transcription (STAT) pathway were involved in regulating gene expression in response to cytokines and growth factors, which was essential for immune response and cell survival. The mitogen-activated protein kinase (MAPK) pathway mediated cellular responses to stress and inflammation, playing a key role in cell growth, differentiation, and apoptosis. Activator protein 1 (AP-1), a transcription factor activated by MAPK, regulates gene expression related to cell proliferation and apoptosis. The nuclear factor kappa-light-chain enhancer of activated B cells (NF-κB) was a critical regulator of immune and inflammatory responses, controlling genes involved in inflammation and cell survival. Polyphenols inhibited these pathways, thereby reducing inflammation, preventing chondrocyte apoptosis, and mitigating joint degeneration [10,11]. Several researchers found that peptides derived from various food sources and agricultural by-products provided strong bioactivities related to inhibiting oxidative stress, scavenging free radicals, and preventing DNA damage [12,13,14,15,16,17]. Due to their superior bioactivity, these peptides have been recognized as promising candidates for natural alternatives to synthetic antioxidants [18,19,20]. Besides the antioxidant activity of peptides, a previous study found that peptides derived from hydrolyzed collagen were absorbed into and retained by cartilage [21]. Chondroitin sulfate was also found to reduce pain in OA and minimize joint space narrowing and cartilage volume loss [22,23].

Eggshell membrane, a naturally protein-rich biomaterial, gained attention as a potential source of bioactive peptides with antioxidant capabilities [20,24]. The antioxidant activity of these peptides was revealed to be enhanced by specific amino acid sequences with particular residues, such as proline (P), histidine (H), tyrosine (Y), valine (V), leucine (L), aromatic side chains (tyrosine (Y), tryptophan (W), phenylalanine (F)), imidazole groups (histidine (H)), and sulfur-containing residues (methionine (M), cysteine (C)) [25]. Clinical studies found that peptides derived from eggshell membranes alleviated symptoms of arthritis due to their antioxidant activity, suggesting that eggshell membranes could be used for OA treatment [26,27,28]. A recent study demonstrated that eggshell membrane has a protective effect against OA in SW-1353 through its antioxidant activity [20]. However, the study lacked profiling bioactive components against cartilage breakdown and specific antioxidant peptides that could contribute to OA prevention.

Thus, the current study initially aimed to identify and quantify bioactive components related to OA prevention, such as hyaluronic acid, collagen, and chondroitin, as well as profile specific antioxidant peptides from the natural eggshell membrane (NEM) utilizing LC–MS/MS. Secondly, the current study measured the degree of protein hydrolysis using the biomimicking in vitro digestion model to examine protein digestibility from the human body. The final purpose was to investigate the protective effect of NEM against OA from human chondrocytes (SW-1353) by measuring expressions of superoxide dismutase (SOD), ROS level, and apoptosis utilizing hyperspectral imaging and live cell imaging techniques.

## 2. Results and Discussion

### 2.1. Profiling of Bioactive Components from NEM

#### 2.1.1. Nutritional Profiling of NEM

Bioactive components from NEM for cartilage, including hyaluronic acid, total sulfated glycosaminoglycans, collagen, and total protein, were quantified, and their content was expressed as percent (%) of sample weight (Table 1). Previous research indicated that external hyaluronic acid (HA) could stimulate HA production in chondrocytes and protect against cartilage breakdown [29]. It was also confirmed that peptides derived from hydrolyzed collagen could be absorbed into and retained by cartilage [21]. Additionally, combining glucosamine with chondroitin sulfate was found to reduce the pain of OA and minimize joint space narrowing and cartilage volume loss [22,23]. Moreover, a randomized, multicenter, double-blind, placebo-controlled clinical trial demonstrated that daily supplementation with 500 mg of NEM^®^ significantly reduced joint pain and stiffness in osteoarthritis patients compared to the placebo group, with improvements observed as early as day 10 and continuing through days 30 and 60 [30]. Results from the current study suggest that NEM could be a good source for protecting human chondrocytes. The present study further profiled peptides due to their relatively high proportion of the total protein in NEM.

#### 2.1.2. Comprehensive Peptide Profiling of NEM via LC–MS/MS Analysis

**Figure 1:**
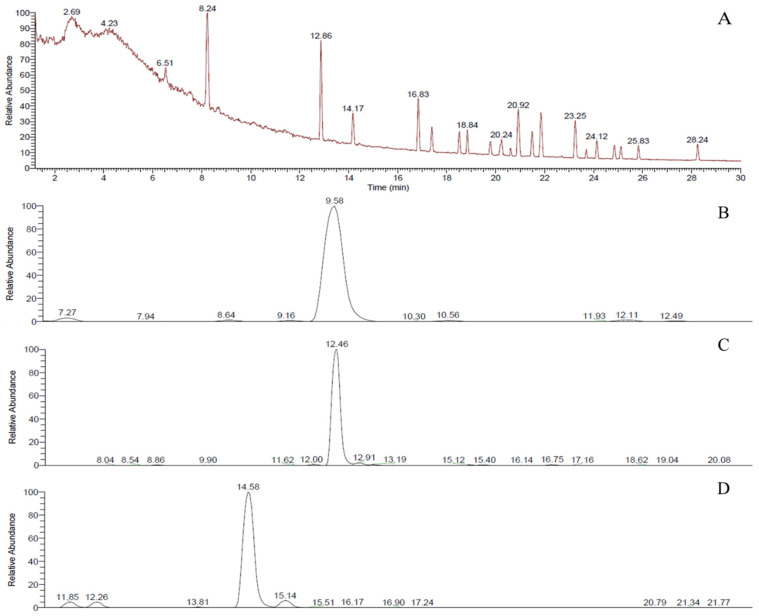
MS chromatogram of NEM (**A**) and extracted ion chromatogram of TEW (**B**), SWVE (**C**), and VYL (**D**) were detected at 9.58, 12.46, and 14.58 min, respectively.

**Figure 2:**
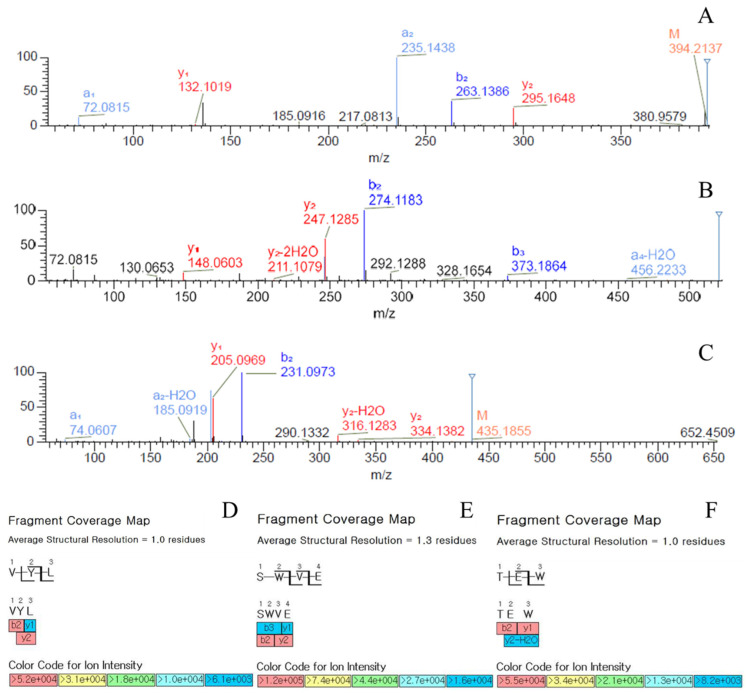
MS/MS spectrum of VYL (**A**), SWVE (**B**), and TEW (**C**) and each fragment coverage map of VYL (**D**), SWVE (**E**), and TEW (**F**).

To identify and quantify peptides from NEM, nano-LC-ESI-MS analysis was performed, and molecular mass analysis using MS MALDI ToF/ToF was confirmed by singly charged ions with an *m*/*z* value corresponding to the theoretical mass of each peptide. The mass ion chromatogram showed distinct peaks corresponding to specific peptides (Figure 1A), and three peptides among them were further identified through sorting parameters (Figure 1B–D). Peaks eluted at 9.58, 12.46, and 14.58 min were found to be Threonine–Glutamic Acid–Tryptophan (TEW), Serine–Tryptophan–Valine–Glutamic Acid (SWVE), and Valine–Tyrosine–Leucine (VYL) peptides with an M/Z value of 435.1874, 520.2402, and 394.2336, respectively (Figure 1). Figure 2 illustrates a comparison of MS/MS spectra for the native forms of the peptides VYL, SWVE, and TEW. The fragment ions are representative, which clearly distinguish the native forms from each other, confirming the high quality of peptide identification. In detail, b-ions formed at the N-terminus of the peptide provide crucial information regarding the early sequence, while y-ions generated at the C-terminus offer insights into the latter part of the sequence. The analysis of these fragmentation patterns of b-ions and y-ions confirmed the ability to accurately determine peptide sequences. The antioxidant activity of these three peptides has not been previously reported in the literature, but the SWVE peptide was characterized from an egg white pepsin hydrolysate and found to have ACE-inhibitory activity [31]. In this study, three novel peptides (SWVE, TEW, and VYL) were identified from NEM. The antioxidant properties of these specific peptides have not been previously reported. A previous study found that hydrolysates from eggshell membrane (ESM) proteins, including peptides like KPLCPP and MDGWPR, were shown to exhibit strong antioxidant and cytoprotective effects, with significant ORAC (Oxygen Radical Absorbance Capacity) values due to the presence of hydrophobic and aromatic residues, which play a crucial role in scavenging free radicals [32]. In a similar manner, the identified peptides in this study (SWVE, TEW, and VYL) contain key residues, such as tryptophan and valine, which are known to enhance antioxidant activity. The structural similarities between these peptides and those reported in previous studies suggest that the NEM-derived peptides may share similar bioactive properties.

#### 2.1.3. Digestive Protein Hydrolysis of NEM Using an In Vitro Digestion Model

**Figure 3:**
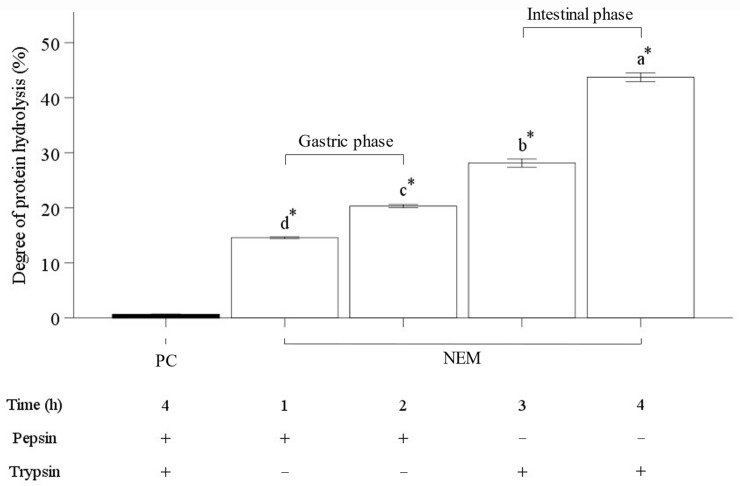
Comparison of the degree of protein hydrolysis of NEM during gastric and intestinal phases using an in vitro digestion model. The vertical bars represent the standard error of the mean of six replications. An asterisk (*) indicates significant differences between the positive control and each treatment group (*p* < 0.05). The different letters indicate a significant difference among groups (*p* < 0.05).

An in vitro digestion model was utilized to measure the extent of amino acids hydrolyzed from NEM protein during the continuous phases of gastric and intestinal digestion (Figure 3). During gastric digestion, 14.57 ± 0.16% and 20.33 ± 0.29% of the protein were hydrolyzed after 1 and 2 h after incubation, respectively. Subsequently, 28.14 ± 0.73% and 43.72 ± 0.76% of the protein were hydrolyzed after 3 and 4 h, respectively, in the intestinal phase. These results indicate that the degree of NEM protein hydrolysis increased progressively over the course of the continuous in vitro digestion. A prior study found a strong correlation between the protein hydrolysis through the pepsin–trypsin in vitro digestion model and in vivo experiments, even though it was in a highly controlled environment with limited endogenous secretion [33]. A recent study on the degree of protein hydrolysis in eggshell membranes using alcalase revealed a hydrolysis rate of 11.70% for fresh eggshell membranes and 15.46% for hatched eggshell membranes [20]. Limited hydrolysis is usually characterized by a low DH value (<10%), while extensive hydrolysis is defined by a high DH value (>10%) [34]. In contrast to common methods that typically measure hydrolysis using alcalase in food studies, the current study focused on hydrolysis through human digestion. This approach demonstrated a higher degree of protein breakdown, suggesting that these peptides could potentially be absorbed more efficiently in the body, thereby increasing their bioavailability, and it is well established that small peptides, consisting of 2–6 amino acids, are efficiently absorbed in the intestine [35,36]. Results from the current study indicated that eggshell membrane proteins were readily hydrolyzed using an in vitro digestion model.

### 2.2. Protective Effect of NEM on Oxidative Stress in Human Chondrocytes

#### 2.2.1. Cell Viability of SW-1353 Treated with NEM

**Figure 4:**
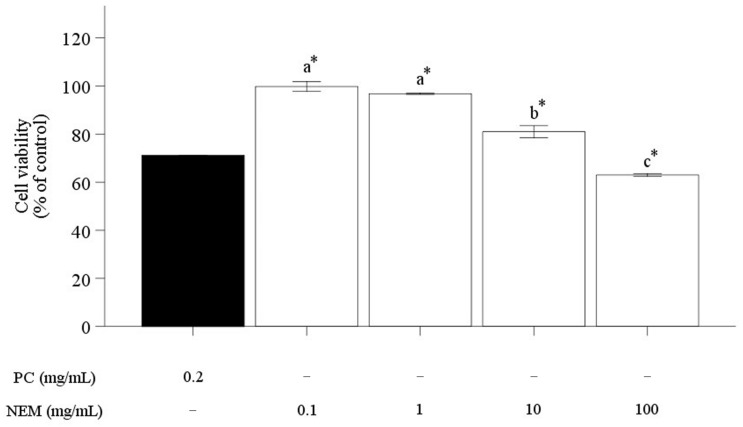
Cell viability (%) of SW-1353 after treating NEM for 24 h. It was normalized by control according to various concentrations. The vertical bars represent the standard error of the mean of six replications. An asterisk (*) indicates significant differences between the positive control and each treatment group (*p* < 0.05). The different letters indicate a significant difference among groups (*p* < 0.05).

The cell viability of human chondrocytes (SW-1353) at positive control (PC) and various concentrations of NEM was assessed using the MTT assay (Figure 4). As the concentration of NEM increased, the viability of SW-1353 decreased in a dose-dependent manner. Its value for PC at 0.2 mg/mL was reached at 71.08 ± 0.12%. The most significant reduction in cell viability after treatment with NEM occurred at 100 mg/mL of concentration, showing 80% of cell viability. Based on these findings, subsequent experiments were conducted using NEM concentrations of 0.1, 1, 10, and 100 mg/mL, which maintain over 80% cell viability.

#### 2.2.2. ABTS Cationic Radical Scavenging Activity of NEM

**Figure 5:**
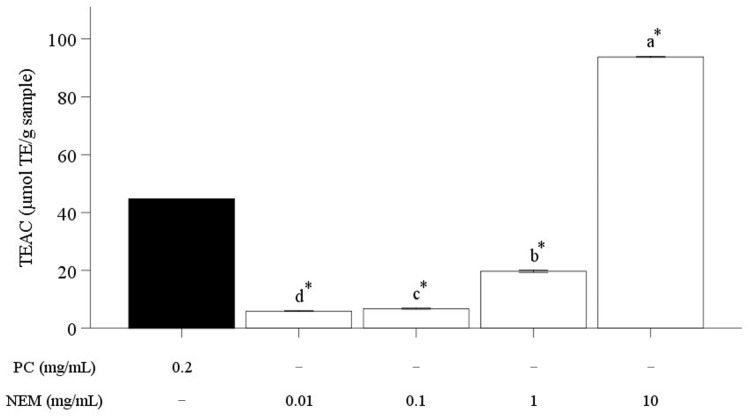
Trolox equivalent ABTS cationic radical scavenging activity (%) of NEM. It was expressed as Trolox equivalent antioxidant capacity (TEAC, μmol TE/g sample). The vertical bars represent the standard error of the mean of six replications. An asterisk (*) indicates significant differences between the positive control and each treatment group (*p* < 0.05). Different letters indicate a significant difference among groups (*p* < 0.05).

The antioxidative capacity of NEM was assessed by measuring its electron donation-mediated radical scavenging activity using the ABTS assay (Figure 5). The ABTS cationic radical scavenging capacity expressed as Trolox equivalent antioxidant capacity (TEAC, μmol TE/g sample) increased in a dose-dependent manner, showing 5.92 ± 0.14, 6.79 ± 0.20, 19.69 ± 0.38, and 93.76 ± 0.23 μmol TE/g sample at concentrations of 0.01, 0.1, 1, and 10 mg/mL, respectively. TEAC of 10 mg/mL of NEM was 2.1 times higher than that of PC (44.72 ± 0.07 μmol TE/g sample), confirming that it possesses the strongest radical scavenging ability among various concentrations of NEM. A similar finding was observed regarding the antioxidant potential of eggshell membranes using assays such as DPPH, Fe^2^⁺-chelating, and reducing power assays [20]. Results from several studies suggest that this antioxidant capacity of eggshell membranes could be derived from antioxidant peptides [26,27,28]. In particular, hydrophobic residues located at the C-terminus or N-terminus of peptide chains, such as V, L, I, A, F, and K, contribute to the stabilization of peptide structures, which enhances their antioxidant properties by allowing them to interact with and neutralize free radicals [16,19,37]. A previous study also reported that aromatic residues (Y, W, F), imidazole groups (H), and sulfur-containing residues (M, C) contributed to the direct electron transfer necessary for neutralizing free radicals, suggesting a strong potential for free radical scavenging activity [38]. Peptides with amino acid sequences such as A, G, P, V, and L have been shown to demonstrate significant antioxidant activity, with Glycine (G) and Proline (P) contributing to the flexibility of the peptide structure, thereby enhancing its ability to effectively interact with reactive species [25]. As shown in Table 2, the current study profiled peptides containing many of these key amino acid residues, including VYL, SWVE, TEW, EKLTEW, HNPTNTIVYF, KSRPILPIYLK, YSPL, IYL, VYLPQ, and SRPILPIYLK, which can provide radical scavenging capacity. Results from this study suggest that NEM-containing peptides have the potential to be a promising natural antioxidant substance. The present study, therefore, further investigated the cellular antioxidant activity of NEM using human chondrocytes (SW-1353).

#### 2.2.3. Superoxide Dismutase (SOD) Expression from SW-1353 with NEM

**Figure 6:**
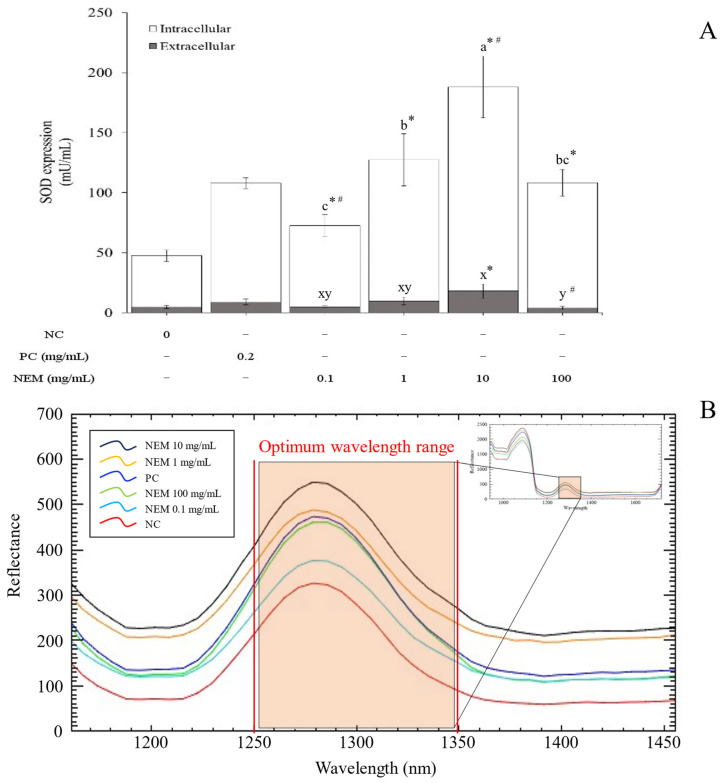
Evaluation of antioxidant efficacy by measuring intracellular superoxide dismutase (SOD) expression in SW-1353 treated with NEM, using a UV–VIS spectrometer at 450 nm (**A**) and hyperspectral imaging (**B**). The vertical bars represent the standard error of the mean of six replications. An asterisk (*) indicates significant differences between the control and each treatment group (*p* < 0.05). A hash sign (#) indicates significant differences between the positive control with different diameters (*p* < 0.05). Different letters indicate a significant difference among groups (*p* < 0.05).

Measurements of SOD expression in SW-1353 after 24 h of NEM treatment are presented in Figure 6A. Intracellular and extracellular SOD expression of PC was 98.97 ± 4.55 and 8.78 ± 2.45 mU/mL, respectively. NEM treatment significantly enhanced both intracellular and extracellular SOD expression at concentrations of 0.1, 1, and 10 mg/mL (*p* < 0.05). Intracellular SOD levels were 67.51 ± 9.33 for 0.1 mg/mL, 117 ± 21.69 for 1 mg/mL, 170.07 ± 25.62 for 10 mg/mL, and 104.49 ± 11.13 mU/mL for 100 mg/mL. Extracellular SOD levels were 4.97 ± 1.24, 9.63 ± 3.30, 18.02 ± 5.80, and 3.80 ± 1.33 mU/mL at 0.1, 1, 10, and 100 mg/mL, respectively. A remarkably high level of both intracellular and extracellular SOD expression was observed at a concentration of 10 mg/mL, suggesting that this concentration represented an optimal threshold for antioxidant defense activation, where NEM-derived peptides potentially provided maximum protective effects against oxidative stress. SOD, the primary defense enzyme against ROS, plays a crucial role in maintaining the balance between oxidation and antioxidation in the body [39]. A similar finding demonstrated that treatment of hatched eggshell membrane hydrolysate for 24 h in SW-1353, followed by treatment with H_2_O_2_, increased SOD1 expression by 1.03-fold and 1.37-fold, respectively, compared to the control [20].

A previous study demonstrated a strong correlation between SOD activity and spectral reflectance values around 1350 nm using partial least squares regression (PLSR), a multivariate regression technique, which highlights the potential for non-invasive monitoring of antioxidant activity based on spectral data [40]. The current study also utilized hyperspectral imaging to predict and confirm SOD activity of NEM (Figure 6B). It was revealed that the peak reflectance near 1350 nm was highest in the order of 10, 1, 100, and 0.1 mg/mL NEM concentrations. This finding correlates with the observed patterns of intracellular and extracellular SOD expression (Figure 6A), indicating that reflectance around 1350 nm could serve as a reliable indicator of SOD activity. Consequently, results from this study suggest that NEM could have the potential to protect against oxidative stress by enhancing SOD expression in SW-1353. The expression of intracellular SOD was commonly assessed using Western blot [20]. Building upon these methods, the present study introduced hyperspectral imaging as a novel approach to predicting SOD activity.

#### 2.2.4. Protective Effect of NEM Against H_2_O_2_-Induced Intracellular ROS Levels and Apoptosis in SW-1353

**Figure 7:**
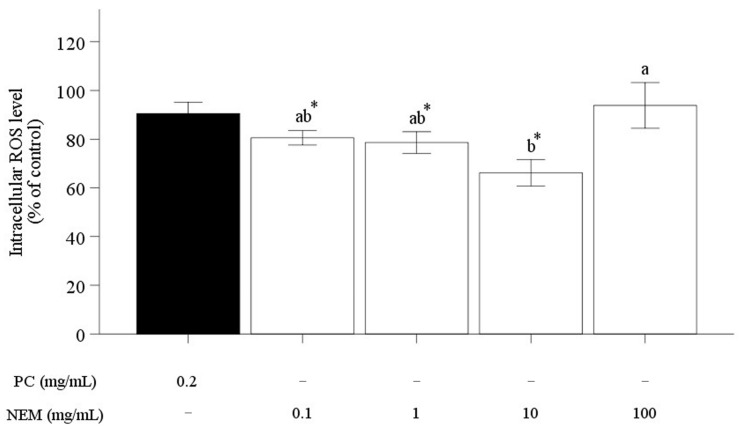
The preventive effect of NEM on intracellular ROS level induced by H_2_O_2_ in SW-1353, as measured by the fluorescence intensity across different groups. It was normalized by control according to various concentrations. The vertical bars represent the standard error of the mean of six replications. An asterisk (*) indicates significant differences between the control and each treatment group (*p* < 0.05). Different letters indicate a significant difference among groups (*p* < 0.05).

**Figure 8:**
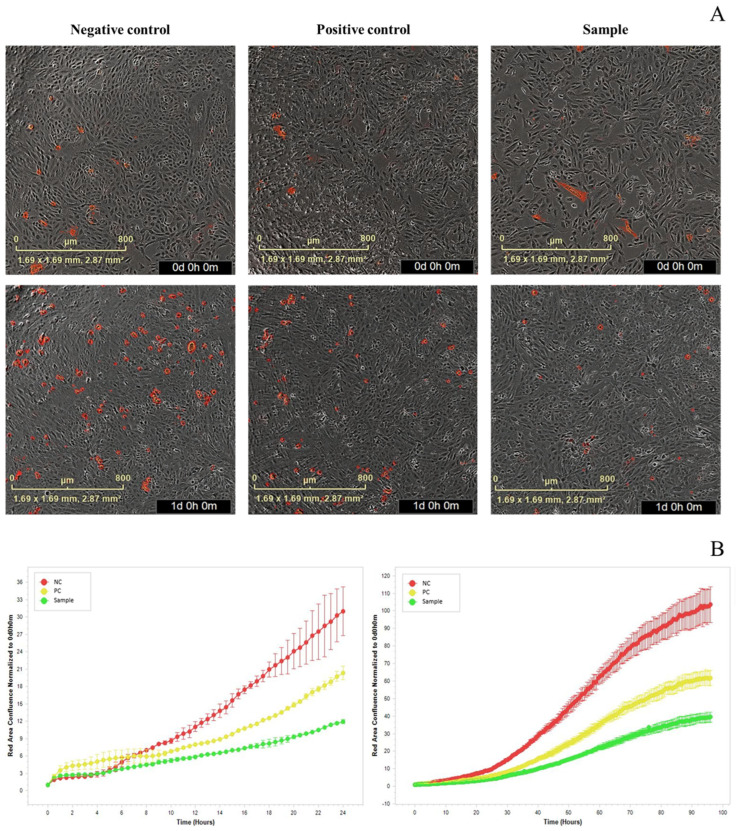
Preventive effect of NEM on apoptosis induced by H_2_O_2_ in SW-1353. (**A**) Red fluorescence of Annexin V micrograph cell imaging for 0 and 24 h; (**B**) Red area confluence normalized to 0 h of different groups for 24 and 96 h.

Oxidative stress levels in cells can be assessed through ROS measurement, and regulating ROS production to inhibit cellular damage in chondrocytes is a critical therapeutic strategy [41]. In this study, ROS levels were determined using DCFH-DA fluorescence dye and then expressed to fluorescence intensity in SW-1353 (Figure 7). Overall, pre-treatment with various concentrations of NEM significantly reduced the fluorescence intensity in SW-1353, which was damaged by the H_2_O_2_ damage group, indicating a decrease in ROS levels induced by H_2_O_2_. Specifically, pre-treatment with NEM at concentrations of 0.1, 1, and 10 mg/mL significantly decreased intracellular fluorescence intensity by 19.47 ± 2.95%, 21.37 ± 4.42%, and 33.82 ± 5.38%, respectively (Figure 7; *p* < 0.05), indicating that NEM can dose-dependently reduce H_2_O_2_-induced ROS levels in SW-1353. The reduction in ROS by PC at a concentration of 0.2 mg/mL was 9.53 ± 4.74%. However, at a concentration of 100 mg/mL, the ROS level increased, which may be associated with the reduction in cell viability observed earlier (Figure 4). Consequently, pre-treatment with NEM appears to provide protection against cellular oxidative stress damage. A comparable study found that pre-treatment with hatched eggshell membrane hydrolysate (0.25, 0.5, and 0.75 mg/mL) significantly reduced the intracellular fluorescence intensity of SW-1353 by 28%, 43%, and 51%, respectively [20]. Another experiment demonstrated that pre-treatment of cells with soluble eggshell membrane protein hydrolysate (SP2), a hydrolysate derived from soluble eggshell membrane protein (SEP), effectively protected against cell apoptosis induced by H_2_O_2_ treatment through downregulation of caspase PARP [24]. Oxidative stress induced by H_2_O_2_ has been confirmed to produce apoptosis as well [42]. To explore whether NEM pre-treatment offers a protective effect against H_2_O_2_-induced apoptosis, the expression of apoptosis was further assessed in cells over a 24 h period using cell imaging. It was revealed that NEM pre-treatment led to a significant reduction in apoptosis expression compared to the control group, indicating a marked suppression of apoptosis (Figure 8A). To further quantify this effect, the red-colored confluence values of apoptosis were normalized to the 0 h mark and monitored over 24 and 96 h using cell imaging (Incucyte^®^ S3, Sartorius^®^, Göttingen, Germany) (Figure 8B). Significant differences were observed among the control, positive control, and NEM-treated groups over the time of incubation (*p* < 0.05). Apoptosis expression was significantly reduced at 10 mg/mL of NEM-pre-treated SW-1353. These findings provide compelling evidence that NEM plays a protective role in cellular pathways related to survival and apoptosis, highlighting its potential to mitigate oxidative stress-related cell death. These findings suggest that NEM may offer a protective effect against cellular oxidative stress, as well as cell apoptosis induced by H_2_O_2,_ by reducing ROS production in chondrocytes. To clarify NEM’s therapeutic potential in oxidative stress treatment, future studies should explore the molecular mechanisms through which NEM regulates ROS and cell apoptosis, and in vivo research is necessary to confirm its effects under physiological conditions.

## 3. Materials and Methods

### 3.1. Chemicals and Reagents

SW-1353 Dulbecco’s Modified Eagle Medium (DMEM) with phenol red, fetal bovine serum (FBS), Dulbecco’s phosphate-buffered saline (DPBS), penicillin/streptomycin (P/S), and trypsin-EDTA were purchased from Biowest Inc. (Riverside, MO, USA). Dimethyl sulfoxide (DMSO), pepsin from porcine gastric mucosa, trypsin from bovine pancreas, hydrochloric acid (6N-HCl), trichloroacetic acid solution (6.1N-TCA), acetone, sodium tetraborate, fluorescamine, 3-[4,5-dimethylthiazol-2-yl]-2,5-diphenyl-tetrazolium bromide (MTT), 2,2′-Azino-bis (3-ethylbenzothiazoline-6-sulfonic acid) diammonium salt (ABTS), potassium persulfate, hydrogen peroxide (H_2_O_2_), and 2′,7′-dichlorofluorescin diacetate (DCFH-DA) were supplied by Sigma-Aldrich Chemical Co. (St. Louis, MO, USA). Formic acid (HCOOH) was purchased from Fisher Scientific (Fair Lawn, NJ, USA), and acetonitrile (ACN), methanol, and HPLC-grade water were obtained from J.T. Baker (Phillipsburg, NJ, USA). The Superoxide Dismutase (SOD) Colorimetric Activity Kit was purchased from Thermo Fisher Scientific Inc. (Waltham, MA, USA). Annexin V was provided by Sartorius^®^ (Göttingen, Germany). All chemicals were of analytical grade and stored according to the specified storage conditions.

### 3.2. Sample Preparation

Natural eggshell membrane (NEM^®^, ESM Technologies, LLC, Carthage, MO, USA) was gifted by the Sung Dong Bio Co., Ltd. (Seoul, Republic of Korea). NEM powder was mixed with 0.1% formic acid and sonicated for 1 min. The solution was then centrifuged at 14,000× *g* for 5 min (ScanSpeed 1580 MGR, LaboGene Co., Ltd., Lillerød, Denmark). The supernatant was filtered through a centrifugal filter unit (Microcon-10 kDa, Merck Millipore Burlington, MA, USA) with a molecular weight cut-off of 10,000 Da.

### 3.3. Analysis of Bioactive Components from NEM

#### 3.3.1. Determination of Hyaluronic Acid Content

The content of hyaluronic acid was determined following the method described in a previous study [43]. A volume of 2.5 mL of sodium tetraborate in sulfuric acid solution was transferred into each tube, followed by the addition of 0.5 mL of sample dilutions. The tubes were then mixed using a vortex shaker for at least 10 s and subsequently heated in a boiling water bath for 10 min. After heating, the tubes were allowed to cool to room temperature. Next, 0.1 mL of carbazole reagent was added to each tube, and the contents were mixed again using a vortex shaker for at least 10 s. The tubes were then subjected to a second heating in a boiling water bath for 15 min, followed by cooling to room temperature. Finally, a microplate reader (Varioskan Flash, Thermo Scientific, CA, USA) was calibrated to zero at 530 nm with no sample in the compartment, and the absorbance of each sample dilution was measured.

$$\%Hyaluronic\;acid=Uronic\;acid\;at\;the\;specified\;dilution\times \frac{Dilution\;factor}{1000}\times \frac{2.16}{10}$$

#### 3.3.2. Determination of Collagen by Hydroxyproline Content

The collagen content was determined using the method described in a previous study [44]. A sample was accurately weighed for 4.0 g and transferred to a 250 mL round-bottom flask with the assistance of a small amount of water. Subsequently, 30 mL of a 3.5 M sulfuric acid solution was added to the flask. The mixture was stirred and heated with a reflux condenser attached for approximately 48 h at an internal temperature of 105 °C. The dark solution was then removed from heat and filtered through filter paper in a vacuum. It was diluted to a final volume of 500 mL with distilled water. From this solution, a 3.0 mL aliquot was further diluted to 100 mL with distilled water.

To prepare for analysis, separate tubes were prepared by adding 2.0 mL of the sample preparation and the hydroxyproline working standards to each tube. An oxidant solution of 1.0 mL was added to each tube, and the mixture was allowed to react at room temperature for 20 min. Following this, 1.0 mL of color reagent was introduced, and the tubes were thoroughly mixed before being heated in a water bath at 60 °C for 15 min. After the heating step, the tubes were promptly cooled in an ice bath for 3 to 6 min. The absorbance of each sample was then measured using a microplate reader at a wavelength of 558 nm. The hydroxyproline content (%) of the test portion, denoted as H, was subsequently calculated.

$$\%H=\frac{h\times 2.5}{m\times V}$$

where

$$h=\frac{Abs-y\;intercept}{slope}$$

$$2.5=Constant=\frac{Initial\;dilution\;volume\times Final\;dilution\;volume}{2\;mL\times (1\times 10^{6}µg/g)}\times 100$$

m = weight of the test portion (g)

V = volume of filtrate used for final dilution (mL)

#### 3.3.3. Determination of Sulfated Glycosaminoglycans (sGAG) Quantified as Chondroitin Sulfate

The determination of chondroitin sulfate was carried out according to the method described in previous research [45]. A sample of 2.00 ± 0.01 g was precisely weighed, along with 242 mg of L-cysteine HCl monohydrate and 212.5 mg of sodium carbonate. These reagents were transferred into a 100 mL round-bottom flask, accompanied by 40 mL of distilled water and a magnetic stir bar. The flask was placed in a heating block or mantle, where the mixture was stirred at 450 RPM and gently heated to 55 °C for 7 min to facilitate acid–base neutralization prior to enzyme addition. Subsequently, 400 mg of alkaline protease was carefully weighed and introduced into the flask. Heating and stirring were maintained for an additional 60 min following the enzyme addition. Upon completion, the flask was removed from the heating block or mantle, and the contents were diluted to a final volume of 500 mL with distilled water.

An aliquot of the sample (40 µL) was transferred into individual semi-micro cuvettes. The cuvettes were then positioned in the instrument, and 960 µL of DMMB-Tris was meticulously added to one of the cuvettes. The absorbance of the solution was recorded at a wavelength of 525 nm, precisely 30 s following the addition of DMMB-Tris. The concentration of sulfated glycosaminoglycans (sGAG) in the sample was subsequently calculated as a percentage using the following equation.

$$\%Total\;sGAG=\frac{\left(Absorbance-y\;intercept\right)}{slope}\times \frac{500}{sample\;weight\left(g\right)\times 10^{6}\;µg/g}\times 100$$

#### 3.3.4. Determination of Protein Content

Protein content was assessed using the method outlined in a previous study [46]. A test portion weighing between 0.250 and 1.000 g was placed into a digestion flask. To this, 15 g of K_2_SO_4_, 0.04 g of anhydrous CuSO_4_, 0.5 to 1.0 g of alundum granules, and 20 mL of H_2_SO_4_ were subsequently added. To verify the accuracy of the digestion parameters, at least one sample of high-purity lysine⋅ HCl was included in each day’s run. The flask was heated at a boil rate for 5 min until dense white fumes cleared the bulb of the flask. The contents were then gently swirled, and heating was continued for an additional 90 min. The flask was allowed to cool, and 250 mL of H_2_O was cautiously added, followed by cooling to room temperature. A titration beaker was prepared by adding an accurately measured volume of standard acid solution along with sufficient H_2_O to ensure that the condenser tip would be adequately immersed. Next, 3 to 4 drops of a methyl red indicator solution (prepared by dissolving 1 g of methyl red sodium salt in 100 mL of methanol) were added. To reduce foaming, 2 to 3 drops of tributyl citrate were introduced into the digestion flask, along with an additional 0.5 to 1.0 g of alundum granules. A NaOH solution prepared by dissolving approximately 450 g of NaOH in H_2_O, cooling, and diluting to 1 L was slowly added down the side of the flask, making the mixture strongly alkaline. The flask was immediately connected to the distillation apparatus, thoroughly mixed, and distilled at a boil rate of approximately 7.5 min until at least 150 mL of distillate was collected in the titration beaker. The excess standard acid in the distillate was titrated with a standard NaOH solution. A blank determination was performed on the reagents, and the nitrogen content (% N) was calculated.

When standard HCl was used:

$$N,\%(w/w)=[\left(M_{acid}\right)(mL_{acid})-({mL}_{bk})(M_{NaOH})-({mL}_{NaOH})(M_{NaOH})][1400.67]/mg\;test\;portion$$

When standard H_2_SO_4_ was used:

$$N,\%(w/w)=[(M_{acid})(2)({mL}_{acid})-({mL}_{bk})(M_{NaOH})-({mL}_{NaOH})(M_{NaOH})][1400.67]/mg\;test\;portion$$

where mL_NaOH_ is the volume of standard base required to titrate the distillate; mL_acid_ is the volume of standard acid used for that distillate; mL_bk_ is the volume of standard base required to titrate 1 mL of standard acid minus the volume of standard base required to titrate the reagent blank carried through the method and distilled into 1 mL of standard acid; M_acid_ is the molarity of the standard acid; and M_base_ is the molarity of the standard base. Finally, the percentage of crude protein was calculated based on the determined nitrogen content.

#### 3.3.5. Peptide Profiling by LC–MS/MS

To analyze sorted peptides in NEM, the analysis was performed according to a previous method [20]. The major peptides in the NEM were sequenced by LC–MS/MS (ESI, MRM) using a UHPLC system (Nexera 40 series, Shimadzu, Tokyo, Japan) with an ACQUITY UPLC Peptide BEH C18 column (130 A, 1.7 μm, 2.1 × 100 mm, Waters, Milford, MA, USA). The loaded samples were eluted with a linear gradient at a flow rate of 0.3 mL/min using 0.1% formic acid in water (A) and 0.1% formic acid in acetonitrile (B) as mobile phases as follows: from 0% to 2% B for 2 min, from 2% to 55% B for 53 min, from 55% to 100% B for 1 min, 100% B for 3 min, from 100% to 2% B for 1 min, and 2% B for 5 min were adopted. MS (Thermo Q-Exactive Plus, Thermo Fisher Scientific Inc., MA, USA) scans were obtained from 300 *m*/*z* to 1800 *m*/*z*. A search of the UniProt Gallus gallus database for data files was performed using Analyst (ver. 1.7.2, SCIEX, Framingham, MA, USA).

Peptide profiling was conducted using the NEM database of Gallus gallus species and alcalase from *Bacillus licheniformis*. Modified peptides were excluded using sorting parameters at a confidence level above 99.0%, and then three final candidate peptides (VYL, SWVE, TEW) containing a minimum of three amino acids were identified. L-Glutamic Acid, L-seryl-L-tryptophyl-L-valyl (SWVE), was selected as the marker substance, and validation tests for the quantification analysis method were performed. The marker component of NEM, which is SWVE, was diluted with 0.1% formic acid in 20% acetonitrile and then measured using LC–MS/MS on a UHPLC system and MS/MS (Thermo Q-Exactive Plus, Thermo Fisher Scientific Inc., MA, USA) in ESI, positive ion mode, MRM methods. Data collection was performed using SCIEX’s Analyst (ver. 1.7.2), and a linear regression with a 1/x^2^ weighting was applied to the calibration curve. Specificity, linearity, accuracy, precision, limit of detection (LOD), and limit of quantification (LOQ) were evaluated using standard solutions of linearity (5, 8, 9, 10, 11, 12, and 15 ng/mL) and test solutions of accuracy and precision at concentrations of 5 (QL), 10 (QM), and 15 (QH) ng/mL. It was calculated as follows:

$$\%Error=\frac{Measured\;concentration-Nominal\;concentration}{Nominal\;concentration}\times 100$$

$$Accuracy\left(\%,recovery\;rate\right)=\left(100-Error\right)$$

The precision was expressed as repeatability, which was the approximation of the results obtained by the same operator, measuring system, conditions, and laboratory in a short time frame. Repeatability was conducted by NEM (5, 8, 9, 10, 11, 12, and 15 ng/mL), and it was replicated three times. The repeatability of the analytical method was calculated using the coefficient of variation (%CV), which is equivalent to the percent of relative standard deviation (%RSD).

$$\%CV=\frac{S.D.of\;measured\;concentration}{Mean\;of\;measured\;concentration}\times 100$$

where S.D. is the standard deviation.

The sensitivity was evaluated using the limit of detection and quantification (LOD and LOQ) determined from the calibration curve.

$$LOD\left(Limit\;of\;dectection\right)=3.3\times \frac{S_{y}}{S}$$

$$LOQ\left(Limit\;of\;quantification\right)=10\times \frac{S_{y}}{S}$$

where S_y_ is the standard deviation of y-intercepts of regression curves, and S is the slope of the calibration curve.

### 3.4. Digestive Protein Hydrolysis of NEM Using an In Vitro Digestion Model System

Digestive hydrolysis of NEM was assessed using an in vitro digestion model system according to the previous method with certain modifications [47]. NEM was mixed with 1 mg/mL pepsin in 10 mM HCl (5%, *w*/*w*, pH 2.0) before placing the mixture in a 37 °C water bath for 2 h to simulate the gastric phase. Then 1 mg/mL trypsin in 1 mM HCl (5%, *w*/*w*, pH 7.6) was added, and the mixture was placed in a 37 °C water bath for 2 h to mimic the intestinal phase. To measure the hydrolysis of NEM, the digestive sample was mixed with an equal volume of trichloroacetic acid solution (TCA 24%), followed by precipitation in ice water for 30 min, and the mixture was centrifuged at 4000× *g* (4 °C, 20 min). After centrifugation, it was mixed with 900 μL of sodium tetraborate (0.1 M, pH 8.0) and 300 μL of fluorescamine acetone solution (0.2 mg/mL) with 30 μL of supernatant. The degree of protein hydrolysis (DH) was used to evaluate NEM digestibility, and DH was determined following a previous work [48]. The fluorescence was measured at an excitation wavelength of 390 nm and an emission wavelength of 480 nm using a spectrofluorometer (Varioskan Flash, Thermo Scientific, CA, USA). The DH was calculated as follows:

$$\%DH=\frac{\left[-NH2\left(t\right)\right]-[-NH2\left(0\right)]}{\left[-NH2\left(\infty \right)\right]-[-NH2\left(0\right)]}\times 100$$

where [−NH2 (t)] indicates the concentration of primary amines after hydrolysis, and [−NH2 (0)] indicates the concentration of primary amines before hydrolysis. The [−NH2 (∞)] was assessed by the fluorescence of each sample (before hydrolysis), which was completely hydrolyzed using 6 M hydrochloric acid at 100 °C for 48 h.

### 3.5. Preventive Effect of NEM on Oxidative Stress in Human Chondrocytes

#### 3.5.1. Cell Culture of Chondrocytes

The SW-1353 chondrocytes were supplied from American Type Culture Collection (ATCC) (Manassas, VA, USA). The cells were cultivated in the DMEM with 10% FBS and 1% P/S at 37 °C in a humidified atmosphere with 5% CO_2_ in 95% air. The medium was refreshed every other day, and it was sub-cultured when confluence reached 70 to 80%. All samples were treated at a confluence up to 90%. All experiments were conducted with a cell passage of under 20.

#### 3.5.2. Measurement of Cell Viability by MTT Assay

The cell viability of SW-1353 was measured using a modified version of the 3-(4,5-dimethylthiazol-2-yl)-2,5-diphenyltetrazolium bromide (MTT) assay, as outlined in a previous study [49]. SW-1353 was seeded with 2 × 10^4^ cells/well in a 96-well cell culture plate. After seeding, the cell was incubated at 5% CO_2_ at 37 °C for 24 h. The cells were treated with DMEM containing various concentrations of NEM (0.1, 1, 10, 100 mg/mL) or with a PC, which was a chondroitin-based supplement matched to the chondroitin content in NEM. Each treatment was incubated separately at 37 °C with 5% CO_2_ for 24 h. After removing the sample solution, 0.5 mg/mL MTT in DMEM was added, and the cells were cultured for 2 h under the same conditions as above. Then, 100 µL dimethyl sulfoxide (DMSO) was added to dissolve the formazan derived from MTT immediately after removing the MTT solution. It was incubated at 37 °C for 10 min. The optical density (O.D.) was measured at 570 nm for the MTT signal and 630 nm for the background by using a microplate reader (Varioskan Flash, Thermo Scientific, CA, USA). The cell viability (%) was calculated by using the following equation:

$$\%Cell\;viability=\frac{Sample\left(O.D\;at\;570\;nm\right)-Sample\left(O.D\;at\;630\;nm\right)}{Control\left(O.D\;at\;570\;nm\right)-Control(O.D\;at\;630\;nm)}\times 100$$

#### 3.5.3. ABTS Radical Scavenging Ability

To investigate the effect of NEM on the scavenging of 2,2′-Azino-bis (3-ethylbenzothiazoline-6-sulfonic acid) diammonium salt (ABTS) radical cation, the ABTS assay was performed according to a previous method [50]. A solution was prepared by mixing 7 mM of ABTS with 25 mL of distilled water, followed by the thorough mixing of 2.55 mM potassium persulfate. The mixture was allowed to react in darkness at room temperature for 16 h. The resulting ABTS solution was then diluted with distilled water until reaching an absorbance of 0.7 ± 0.05 at 734 nm. Subsequently, the ABTS working solution (180 µL) and samples (20 µL) were added to a 96-well plate, and the reaction was conducted at room temperature in the dark for 5 min. It was measured at 734 nm for the ABTS radical scavenging signal using a microplate reader. The ABTS radical scavenging ability (%) was calculated using the equation provided below:

$$\%ABTS\;radical\;scavenging\;ability=1-\frac{Sample\left(O.D\;at\;517\;nm\right)}{Control\left(O.D\;at\;517\;nm\right)}\times 100$$

#### 3.5.4. Intracellular Superoxide Dismutase (SOD) Expression from SW-1353 with NEM

SW-1353 was seeded with 1 × 10^6^ cells/well in a 6-well cell culture plate. After seeding, the cells were incubated at 5% CO_2_ at 37 °C for 24 h. Cells were treated with various concentrations of NEM for 24 h. The cells in phosphate-buffered saline were homogenized by sonication and clarified by centrifugation at 1500× *g* for 10 min at 4 °C. The expression of SOD in cell homogenates was determined using a SOD colorimetric activity kit from Thermo Fisher Scientific Inc. (Waltham, MA, USA), which utilizes a tetrazolium salt for the detection of superoxide radicals generated by xanthine oxidase and hypoxanthine at 450 nm. SOD expression was determined from a standard curve, and specific activity was obtained by dividing SOD expression by protein concentration.

#### 3.5.5. Superoxide Dismutase (SOD) Activity Using Hyperspectral Imaging

A hyperspectral camera (Specim FX17, Geostory, Oulu, Finland) ranging from 900 to 1700 nm wavelength was used for taking images of SOD activity from chondrocytes, and then each image was divided into 112 bands. Spectral imaging software (Lumo scanner, Middleton Spectral Vision, Middleton, WI, USA) was used to capture the images, and after the shooting, normalization was conducted to subtract the black-and-white values. The base signal of the detector (black reference) was the reflection value taken in a dark state, and the spectral range (white reference) was the reflection value of the white bar, which served as the standard for the sample position when taking pictures. Two values were removed through normalizing because they can affect the reflection values. Through this process, the HSI of each sample was obtained. Reflectance values from the hyperspectral images were extracted using ENVI 5.2/IDL 8.4.1 software (Harris Geospatial Solutions, Boulder, CO, USA). All of the data were followed by operations.

1. Raw images were normalized by removing black reference, white reference, and noise.
2. Using the region of interest (ROI), samples were designated and identified, and reflectance values were extracted in all ranges of wavelength.
3. A reflectance value graph at a specific wavelength was presented, and the mean reflectance values for each sample were derived for statistical analysis.

Through this process, spectral preprocessing was applied, and the degree of protein content in each sample was determined using a correction coefficient. The protein content in the samples is presented through reflectance value graphs, and it could enable rapid prediction of protein content.

#### 3.5.6. Preventive Effect of NEM on the Intracellular ROS Level and Apoptosis Induced by H_2_O_2_ in SW-1353

A slightly modified version of the method described in a previous study was used to measure the preventive effect of NEM on ROS levels induced by H_2_O_2_ [51]. SW-1353 was seeded into 96-well plates (2 × 10^4^ cells/well), and various concentrations of NEM were pre-treated for 2 h before treating H_2_O_2_. The cells were exposed to 1 µM H_2_O_2_ for 24 h and then were washed with PBS solutions. A 10 μM 2′7′-DCFH-DA solution was added into each well for 30 min. Fluorescence was measured at 488 nm for excitation and 525 nm for emission using a microplate reader (Varioskan Flash, Thermo Scientific, CA, USA). The protective effect of NEM on H_2_O_2_-induced ROS was calculated as follows:

$$Intracellular\;ROS\;level\left(\%\;of\;control\right)=\frac{Sample\left(\mathrm{EX:}\;488/\mathrm{EM:}\;525\;nm\right)}{Control\left(\mathrm{EX:}\;488/\mathrm{EM:}\;525\;nm\right)}\times 100$$

The protective effect of NEM on H_2_O_2_-induced apoptosis was analyzed by cell imaging for 24 h (37 °C, 5% CO_2_) and by measuring the red area confluence using cell imaging (Incucyte^®^ S3, Sartorius^®^, Göttingen, Germany).

### 3.6. Statistical Analysis

All the experiments were performed in six iterations, and the results are presented as the means ± standard deviation (SD). A one-way analysis of variance (ANOVA) was performed to measure significant differences between the groups at the significant value of *p* < 0.05 using SPSS for Windows (Release 18.0, SPSS Inc., Chicago, IL, USA). In addition, 50% inhibitory concentrations (IC_50_) for cell viability, DPPH radical scavenging ability, elastase inhibitory activity, and melanin content inhibition were calculated using the probit method in SPSS for Windows.

## 4. Conclusions

This study highlights the physicochemical properties of NEM and its significant protective effects against H_2_O_2_-induced oxidative stress in human chondrocytes. NEM was found to possess a high content of essential bioactive compounds, including hyaluronic acid, sulfated glycosaminoglycans, and collagen, all of which contribute to its potential health benefits for cartilage. In vitro digestive hydrolysis demonstrated that NEM was well hydrolyzed under the human gut system. Profiling of peptides was revealed to have bioactive peptides VYL, SWVE, and TEW, which are known to provide antioxidant potential. NEM exhibited substantial radical scavenging activity, effectively reducing intracellular reactive oxygen species (ROS) and enhancing superoxide dismutase (SOD) expression. Pre-treatment of NEM mitigated apoptosis in SW-1353 and demonstrated its protective role in oxidative stress environments. In particular, the concentration of 10 mg/mL was found to be the most effective across all experiments, showing significant antioxidant activity and protective effects against oxidative stress. Taken together, the findings suggest that NEM holds promise as a functional ingredient in combating oxidative stress and promoting cartilage health, particularly in conditions related to cartilage degradation and joint disorders. Future research should focus on elucidating the molecular mechanisms underlying these protective effects and evaluating NEM’s therapeutic potential in vivo.

## Figures and Tables

**Table 1:** Content of bioactive components from NEM. The values represent the mean ± standard error (SE) of six replicates.

Bioactive Component	Content (%)
Hyaluronic acid	1.1 ± 0.07
Total sulfated glycosaminoglycans as chondroitin sulfate	1.2 ± 0.25
Collagen	3.1 ± 0.33
Total protein	54.4 ± 2.40

**Table 2:** Sorted peptides of NEM were selected based on the following sorting parameters: a confidence score of ≥99.0%, a minimum of 3 amino acids, positive UV absorbance, peptides from ovalbumin-related protein X, and MS area values.

No.	Peptide Sequence	Delta (ppm)	Wavelength (nm)	Confidence Interval (%)	Retention Time (min)	Z	*m*/*z*	MS Area
1	VYL	0.70	214, 254 and 280 nm	99.8	14.57	1	394.2336	633,690
2	SWVE	0.93		99.8	12.44	1	520.2402	522,964
3	TEW	0.34		99.9	9.55	1	435.1874	133,194
4	EKLTEW	0.38		99.4	15.34	2	403.2081	94,455
5	HNPTNTIVYF	0.59		100.0	22.59	2	603.3011	67,261
6	KSRPILPIYLK	0.64		99.9	21.13	3	443.2868	62,447
7	YSPL	1.19		100.0	13.56	1	479.2500	43,327
8	IYL	0.07		99.1	18.70	1	408.2493	41,438
9	VYLPQ	0.39		100.0	15.53	2	310.1761	35,395
10	SRPILPIYLK	0.20		100.0	24.01	3	400.5885	20,484

## Data Availability

The original contributions presented in the study are included in the article, further inquiries can be directed to the corresponding author.
